# Design and Iterative Development of Serious Exergames for Children With Autism Spectrum Disorder: Formative Multiple-Case Pilot Study

**DOI:** 10.2196/77727

**Published:** 2026-03-02

**Authors:** Won Kim, Minwoo Seong, SeungJun Kim

**Affiliations:** 1 Department of AI Convergence Gwangju Institute of Science and Technology Gwangju Republic of Korea; 2 Department of Computer Engineering College of IT Convergence Chosun University Gwangju Republic of Korea

**Keywords:** autism spectrum disorder, children, design research, exergame, physical activity, serious game

## Abstract

**Background:**

Children with autism spectrum disorder (ASD) exhibit cognitive, motor, and social difficulties that affect engagement, causing developmental delays, behavioral challenges, and obesity—interrelated concerns in daily functioning and well-being. Although interactive interventions have incorporated physical activity, they often rely on limited physical involvement and lack iterative, expert-informed design, as built on pre-existing game frameworks. Physical activity is often operationalized as constrained input (eg, gestures or in-place actions) rather than exertion-intensive, whole-body exercise, and design guidance for adapting exercise content under ASD-oriented safety and cognitive-sensory constraints remains limited. These limitations highlight the need for exergames that promote sustained, full-body participation aligned with developmental goals, motivating formative, co-design with expertise and initial field testing in this population.

**Objective:**

We aim to iteratively design exercise-based serious games (SGs) for children with ASD through a structured, expert-informed co-design process involving 21 professionals across special education, adapted physical education, and human-computer interaction, and to examine feasibility and use contexts through an exploratory multiple-case pilot study.

**Methods:**

We derived serious exergames using 4 design methods—stakeholder interview, concept mapping, creative matrix, and visualize the vote. Two exergames—“Fruit Sorting Run” and “Hazard Avoiding Ride”—were developed, integrating full-body running and cycling movements into goal-directed tasks under ASD-oriented constraints. We conducted a multiple-case pilot with 3 children with ASD. During gameplay, caregivers labeled engagement using a binary input interface, and we conducted postsession caregiver interviews to capture complementary observations.

**Results:**

Engagement in both exergames tended to increase over normalized time. Generalized estimating equations with a logit link and an autoregressive working correlation of order 1 (AR1), including participant indicators, showed a statistically significant association between normalized time and engagement in Fruit Sorting Run (per 0.1 increase: β=0.48; odds ratio 1.62, 95% CI 1.09-2.38; *P*=.02) and Hazard Avoiding Ride (per 0.1 increase: β=0.66; odds ratio 1.93, 95% CI 1.04-3.60, *P*=.04). Caregiver interviews reinforced these findings, reporting increased attention, motivation, and enjoyment across both activities.

**Conclusions:**

The findings support the applicability of an expert-informed design approach and the viability of the resulting exergames, integrating goal-directed physical activity, virtual agent–based prompting, and stakeholder-informed considerations such as motor-cognitive alignment, interactive scaffolding, and support for daily living skills. Distinct from prior SG approaches that operationalize physical activity through discrete gestures or in-place interactions, the proposed exergames embed sustained, exertion-intensive, whole-body movement within structured gameplay. Within this exploratory multiple-case pilot, engagement trajectories tended to increase over time. These preliminary observations provide an initial basis for a testable hypothesis that exertion-intensive, full-body SGs with virtual agent–based prompting may be associated with increasing engagement over time, meriting further examination in larger samples and applied educational and therapeutic contexts.

## Introduction

### Background

Autism spectrum disorder (ASD) is a neurodevelopmental condition characterized by cognitive, motor, and social difficulties [1], which affect daily functioning [2] and overall well-being [3]. Reduced participation highlights the importance of active engagement in supporting adaptive functioning and skill acquisition. A lack of active engagement may lead children with ASD to experience further developmental delays [4], increased risk of stereotyped and challenging behaviors [5], and a higher incidence of obesity [6]—all of which are interrelated and pose significant concerns. Given the scope of these difficulties, the continued rise in ASD diagnoses over the past decades underscores the importance of creating accessible interventions to support active participation in everyday contexts [7]. For example, serious games (SGs) have been explored as digital interventions that target functional outcomes beyond entertainment [8,9], including social communication, executive functioning, and motor coordination [10,11]. Recent SGs have broadened interaction design through modalities such as motion-tracking [12-15], interactive stimuli [16-18], extended reality [14,19], and socially assistive robots [20-23] or virtual agents (VAs) [24,25], enabling diverse forms of feedback and goal-directed activities [26-28]. Within this design space, SG interventions for children with ASD have positioned movement-based physical activities as a central design emphasis to support active participation.

### Related Works

#### Constrained Physical Interaction in Exergames

While prior interactive systems for children with ASD have incorporated physical activity, often using motion-tracking technologies to facilitate interaction, these have primarily involved constrained forms of bodily input or static posture—particularly basic motion gestures [13,14,17,26,28-30] as well as touch [24] and button presses [18]. For example, Bhattacharya et al [26] used Kinect to design motion-based activities to engage students with ASD in a classroom setting by encouraging them to make specific movements or gestures to animate elements of a story on a screen. To improve eye-body coordination, Caro et al [28] proposed the FroggyBobby game, which used Kinect motion-tracking to control the upper limbs as the tongue of Frog and catch as many flies as possible through limb movements. Bossavit et al [27] presented a natural user interface SG that supported movement-based interaction for high-functioning children with ASD [27]. Similarly, Pena et al [29] introduced “Circus in Motion,” a multimodal exergame designed to support vestibular therapy through in-place actions involving the head, upper limbs, lower limbs, and jumping movements for people with ASD. These systems relied on in-place movements of the upper or lower limbs, using predefined gesture-based inputs that were stylized or scripted, and structured in ways that may limit the continuity and spontaneity of physical involvement, revealing a gap in current approaches. This study adopts full-body exercises to extend beyond discrete interaction gestures—short, predefined inputs involving single-limb or in-place movements—by embedding physical activities into gameplay through whole-body movements designed to be accessible to children with ASD.

#### Toward Exertion-Intensive Serious Game Design

As supporting evidence, studies in behavioral intervention have demonstrated that higher-intensity physical activity, even when delivered as simple exercises like jogging or walking, can enhance engagement and reduce stereotyped behaviors among children with ASD. For example, Nicholson et al [31] reported improved on-task behavior following antecedent jogging sessions, and Neely et al [32] found that exercise until satiety produced the greatest behavioral benefits compared with no or brief exercise. These findings suggest that more rigorous and sustained physical activity may have specific benefits for attention and behavior regulation, highlighting the need to design interactive systems that embed such activity within playful and intrinsically motivating formats, such as exertion-intensive SGs, to enhance both engagement and functional outcomes for children with ASD. While some studies have incorporated task-oriented physical activities, these interventions were primarily designed for vocational purposes targeting adolescents or young adults, such as the “Mopping Game,” which used mixed reality to support vocational training [14]. Other examples, such as “PuzzleWalk,” or commercial games like “Pokémon GO [33],” combined walking with pre-existing games and primarily targeted adults [15,34], therefore often lacking iterative expert involvement and offering limited flexibility beyond pre-existing game frameworks. In addition, designing exertion-intensive SGs for children with ASD requires careful selection, adaptation, and progression of exercises to balance capability and cognitive–sensory tolerance while maintaining safety constraints. Design guidance on exercise content for SG-based interventions in this population remains limited [35], motivating a formative co-design process with special education and adapted physical education (APE) professionals.

### Objective

The primary objective of this study is to iteratively design serious exergames through a structured co-design process involving professionals from multiple disciplines, including special educators, APE therapists, and human-computer interaction (HCI) researchers. Building on a series of expert interviews and collaboration, the study focuses on exergame development from the initial selection of exercises to the progressive shaping of gameplay. This process spans conceptualization, design, and development, and aims to support active participation and functional development in children with ASD. To explore feasibility and use contexts, we conducted an exploratory multiple-case pilot with 3 children with ASD, focusing on case-based description of engagement and interaction patterns.

## Methods

### Ethical Considerations

This study involving autistic participants was conducted following the ethical guidelines and regulations of the Institutional Review Board at Gwangju Institute of Science and Technology (approval number HR-61-04-04). The approved protocol includes the involvement of professionals from multiple disciplines and the participation of children with ASD and their legal guardians (eg, parents).

Participant recruitment was conducted in accordance with Institutional Review Board guidelines. The study was introduced at the “Dream Tree Children Education Center” by therapists; 4 parents voluntarily enrolled their children for the experiment. Prior to participation, the experimental procedure and the role of the proxy user were explained to the parents, and informed consent was obtained. Additionally, each parent provided written assent, acknowledging their voluntary participation and the use of their data for research. They were informed that the experiment could be discontinued at any time upon request by the child, guardian, or therapist. The experiment commenced upon confirmation of the child’s willingness to participate, and participants received monetary compensation equivalent to approximately US $70.

To maintain confidentiality, all data were deidentified immediately upon collection. Unique identification codes were assigned to each participant, and personally identifiable information was stored separately in a secure, password-protected database, with access restricted to the primary researcher.

### Interdisciplinary Co-Design

#### Design Considerations

**Figure 1:**
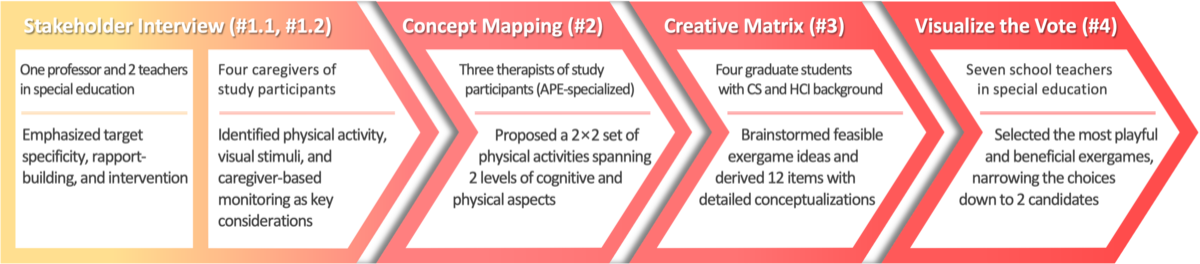
Iterative design process to derive exergame candidates, progressing from initial characterization of children with autism spectrum disorder (ASD) to exergame conceptualization and final expert-based selection, as indicated by the arrow direction and increasing color intensity. APE: adapted physical education; CS: computer science; HCI: human-computer interaction.

This study involved collaborations with multidisciplinary experts, with participation formats—ranging from semistructured interviews to group sessions—structured around the needs of each phase. Experts were recruited from a pool of professionals who expressed interest in related local research programs (Table 1) and were selected based on their specific domain expertise to facilitate concept refinement, activity framing, and final selection (Figure 1).

To formulate exergames tailored for children with ASD, we applied the LUMA framework, which offers structured methods for synthesizing expert-derived insights into actionable design decisions [36]. Stakeholder interview—a design method for eliciting field-informed input through direct dialogue—was used to refine initial design directions. Concept mapping was then applied to organize therapist-proposed exercises by aligning cognitive and motor capabilities. Creative matrix, a method for generating ideas at the intersections of exercise and game features, was used to conceptualize exergames. Then, visualize the vote, a method for polling collaborators to reveal preferences and opinions, was used to select exergame concepts.

**Table 1:** Expert configuration across the study process, with numbers representing the composition of experts (n=21) recruited at each stage of the multistage exergame design for children with autism spectrum disorder (ASD).

Stage	Expert group	Expertise (average years of affiliation)	Collaboration type	Expert role
1.1	Professor (n=1); teachers (n=2)	Special education (>20)	Onsite semistructured interview (individual)	ASD heterogeneity, design refinement
1.2	Caregivers (n=4)	Caregiving expertise (>5)	Onsite semistructured interview (individual)	Refining intervention elements
2	Therapists (n=3)	APE^a^ (>5)	Online semistructured interview (as a group)	Framing APE content
3	Researchers (n=4)	Computer science/HCI^b^ (>7)	Practitioner ideation (as a group)	Exergame conceptualization
4	Teachers (n=7)	Special education (> 15)	Online semistructured interview (Individual)	Final exergame selection

^a^APE: adapted physical education.

^b^HCI: human-computer interaction.

#### Design Method 1: Stakeholder Interview

To address the heterogeneity of ASD diagnoses across regions and to refine the initial game design approach, we conducted individual interviews (n=3) with a professor in special education and 2 special education teachers (>20 years of experience). The first expert interview indicated that the broad spectrum of disabilities necessitates specifying target groups or focusing on specific participants (eg, symptom, degree, and age), suggesting a case study approach. The interview also highlighted the importance of designing experiences that foster a sense of achievement through engagement, particularly by building rapport when introducing unfamiliar characters, game elements, or devices. Additionally, experts discussed the role of rapport and the potential use of virtual or realistic agents. To enhance interaction and motivation, they emphasized the incorporation of promptings and reinforcement feedback to support participation for children with low motivation.

The second interview with the caregivers of our study participants (n=4) highlighted attentional challenges in children with ASD, emphasizing the need for interventions that incorporate physical exertion and visual stimuli (eg, interactive elements) to sustain engagement across varying levels of symptom severity. In particular, these elements were identified as critical for maintaining attention and eliciting curiosity. The caregivers also noted that children with ASD often exhibit emotional fluctuations and that facial expressions and behaviors may not reliably reflect their internal emotional states (eg, a child may smile despite experiencing anxiety). Given these considerations, the interviews underscored the importance of monitoring engagement through observers with an in-depth understanding of the child’s behavioral cues, such as caregivers, to more accurately interpret emotional and attentional states.

#### Design Method 2: Concept Mapping

**Figure 2:**
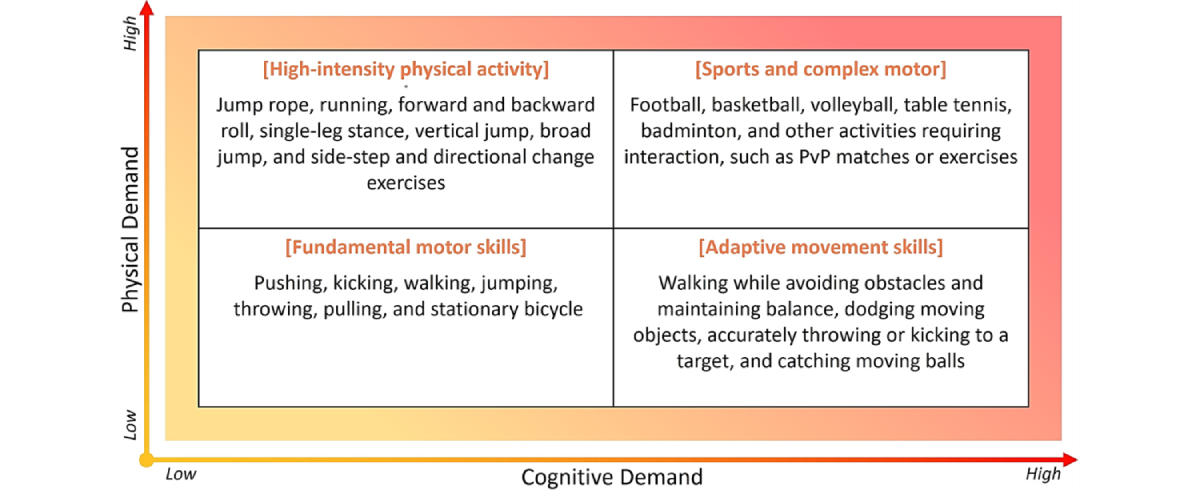
Therapist-proposed exercises serving as the basis for exergame design, organized in a 2×2 matrix according to cognitive and physical demands (n=3). Cognitive demand increases along the horizontal axis, and physical demand increases along the vertical axis. PvP: player vs player.

We collaborated with therapists (n=3) who have expertise and experience (>5 years) in APE to derive readily applicable physical activities tailored to children with ASD participating in this study. These experts were APE specialists practicing at local centers, selected to reflect the practical constraints and requirements of the target environment. The therapists proposed a set of exercises that the participants were likely to recognize and engage with. To incorporate both cognitive and physical aspects, they organized these activities within a 2×2 framework, which served as a foundation for identifying and refining exergame ideas (Figure 2). This framework guided exercise selection for children with ASD, which subsequently structured a brainstorming session to develop exergame concepts for implementation.

#### Design Method 3: Creative Matrix

**Figure 3:**
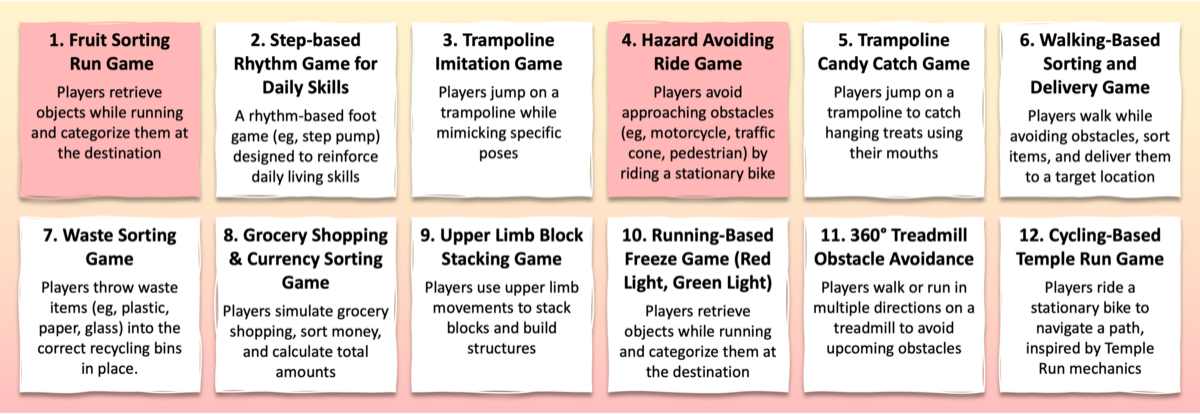
Brainstormed exergame concepts generated through practitioner ideation based on adapted physical education (APE) and human-computer interaction (HCI) expertise, with concepts 1 and 4 selected as final candidates through a voting process involving special education teachers (n=7).

Based on suggestions of physical activities spanning low to high physical and cognitive loads, graduate students (n=4) with an HCI background brainstormed feasible exergames that integrate cognitive and physical demands. The criteria included embedding physical activity scenarios, ensuring accessibility for children aged approximately 10 years, maintaining feasibility for children with ASD, and supporting daily living skill improvement. We derived 12 exergames along with their detailed conceptualizations and prioritized them through a voting process involving special education teachers (Figure 3).

#### Design Method 4: Visualize the Vote

The final selection process involved special education teachers (n=7) and focused on the following two aspects (1) playfulness, ensuring that children with ASD can actively participate, and (2) the potential developmental benefits of the game, such as supporting daily living skills. Each teacher was asked to select up to 3 exergames they considered the most playful SGs. Two exergames—“Fruit Sorting Run” (1) and “Hazard Avoiding Ride” (4)—received the highest number of votes (5 and 4, respectively) and were the only ones selected by all participants.

### Design Principles and Exergame Development

#### Overview of the 2 Serious Exergames

**Figure 4:**
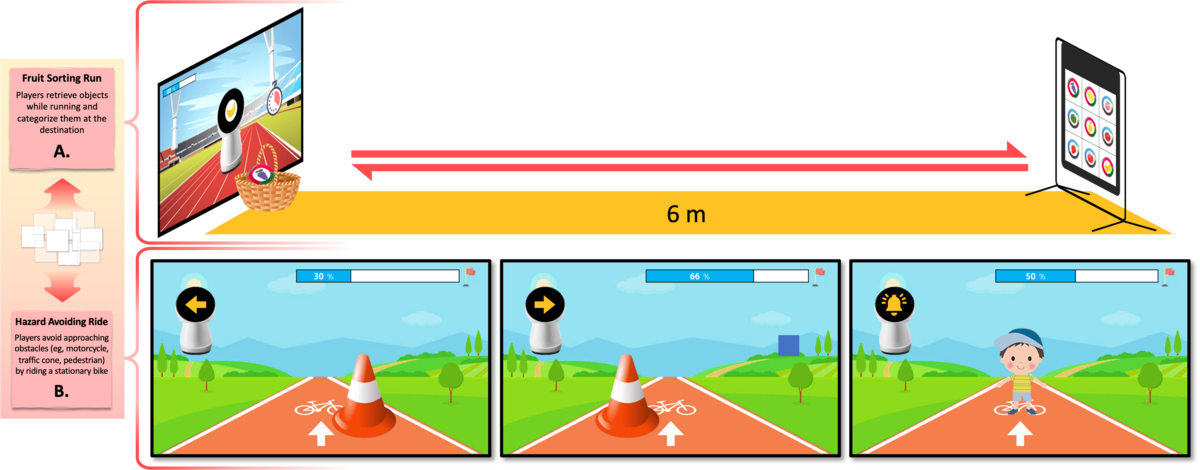
Illustrations of the developed exergames based on running and bicycling, depicting (A) the Fruit Sorting Run game and (B) the Hazard Avoiding Ride game, along with their spatial setup and representative in-game interfaces.

Through an iterative design process involving multidisciplinary expertise, we derived and finalized 2 exergames: Fruit Sorting Run and Hazard Avoiding Ride (Figure 4). Rooted in running and bicycling activities, these games integrate full-body dynamics with daily living skills training in accordance with SG design principles. The resulting system supports exertion-intensive gameplay under ASD-oriented constraints through VA-mediated scaffolding and audiovisual feedback for immediate reinforcement [37] within an animated game environment [24,35]. To prioritize predictability and minimize cognitive load, difficulty levels are kept constant; this approach enables children to focus on functional task performance—fruit categorization and hazard avoidance—while mitigating potential anxiety associated with increasing difficulty [38]. The system was implemented using the Unity 2D engine and refined through iterative adjustments following an initial evaluation session involving a therapist and a child with ASD.

#### Exergame 1: Fruit Sorting Run

**Figure 5:**
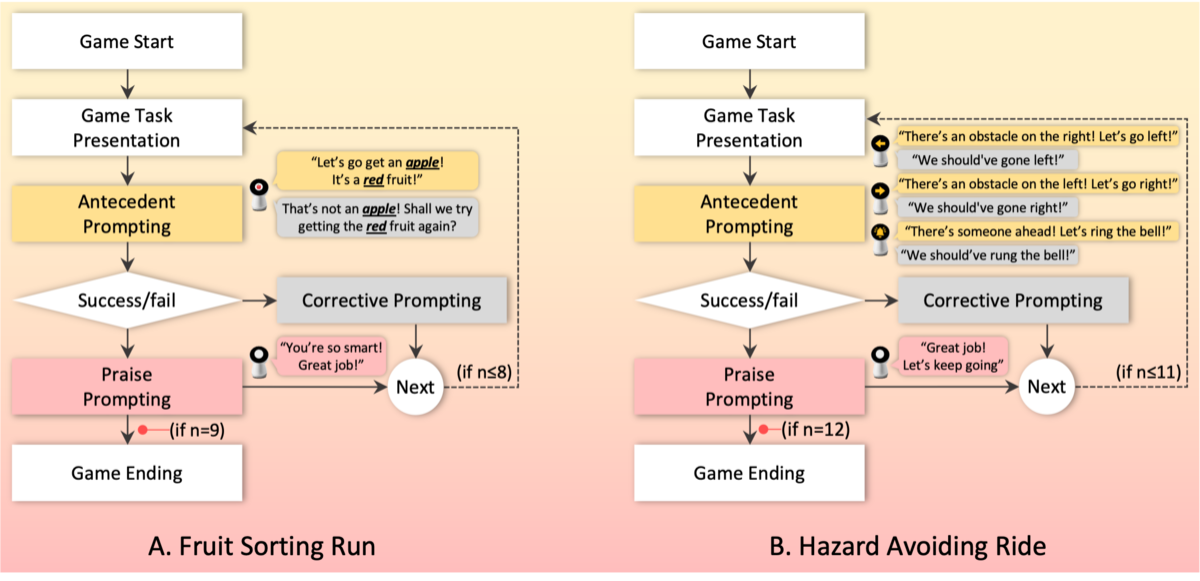
Game logic and structured prompting flow for the 2 developed exergames, illustrating how tasks progress across stages and branch according to success or failure, while different prompting strategies are applied conditionally throughout gameplay; antecedent prompts appear earlier in the flow, corrective prompts are introduced following unsuccessful attempts, and praise prompts accompany successful task completion, each distinguished by a consistent color scheme.

This game aims to enhance visual-perceptual abilities by having children classify velcro balls, each labeled with a fruit image, while walking back and forth across a 6 m straight area (Figure 4A). The game starts in a static game scene where the VA appears at the center of the screen in the running track background. The child observes a fruit image presented on the VA’s screen, runs to the back wall to retrieve a ball with the corresponding image, and places it in a basket positioned below the screen. A total of 9 velcro balls are used, each depicting a different fruit (eg, peach, grape, apple, strawberry, tomato, banana, watermelon, oriental melon, and lemon), and all are randomly placed on the sticky wall. The child is tasked with collecting 9 randomly presented fruit images within a given time limit. The game ends when all balls have been transferred and the progress bar is completely filled. The exergames developed in this study were structured according to the following game logic (Figure 5A).

#### Exergame 2: Hazard Avoiding Ride

This game aims to enhance visual-perceptual abilities by having children ride a stationary bicycle while avoiding obstacles that appear on the left or right side of the VA’s screen through body tilting. Three types of events are randomly presented: (1) a traffic cone on the left, (2) a traffic cone on the right, and (3) a pedestrian ahead (Figure 4B). Participants are required to tilt their bodies in the opposite direction of the obstacle (eg, to the right when a traffic cone appears on the left, and to the left when it appears on the right). When a pedestrian appears in front of the player, the participant must press a bell attached to the bicycle to signal a warning. The game consists of 12 sessions and ends when the progress bar is fully filled according to game logic (Figure 5B).

#### Virtual Agent Integration

To facilitate sustained engagement and provide prompting support within exergames, a VA was integrated into the system. The VA was modeled after Jibo [23], a human-like robot with a head and torso [37,39] that was used to build rapport with children with ASD before gameplay [24,25]. During gameplay, the VA appeared in the game scene and delivered prompts aligned with 3 established categories [40]. These prompts included not only verbal instructions but also visual cues displayed on the VA’s screen to guide task execution. Specifically, antecedent prompting involved directive cues presented before task initiation to guide the child’s actions (eg, “Let’s go get an apple”). Corrective prompting was provided after an attempted response to help the child adjust or refine their behavior (eg, “That’s not an apple! Shall we try getting the red fruit again?“). Praise prompting consisted of positive reinforcement to encourage continued engagement throughout the tasks (eg, “Great job!” and “Yes, that’s correct”). In each gaming context, prompting strategies were tailored based on the task flow and game-specific mechanics of each exergame (Figure 5).

#### Adjustments Following Initial Evaluation

A preliminary study with a child with ASD (a boy, aged 11 years) and one therapist highlighted the need for explicit background audio, auditory prompting, and causal feedback; the volume of prompting and game-related sound effects was increased. Feedback for the final stage was added to support a sense of completion (eg, flag or applause). The study also identified the importance of a progress bar, which enabled the child to track both task progression and their own pace [38]. The progress bar was filled as each session ended. For the Hazard Avoiding Ride, the original 24-session version was reduced to 12 sessions to keep the total duration within 3 minutes, which applies to both exergames.

### Exergame Setup

**Figure 6:**
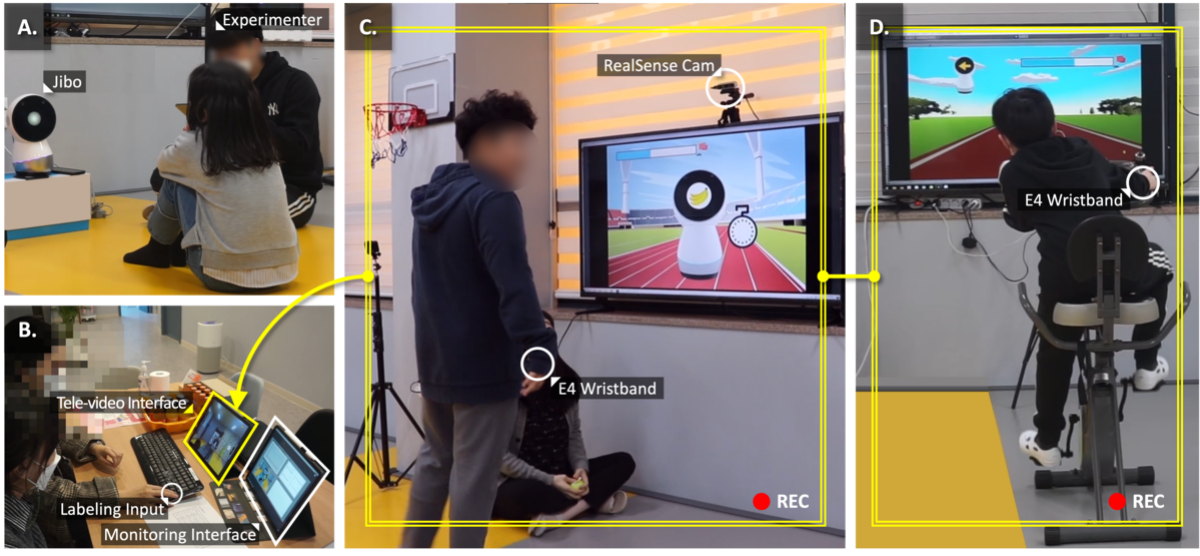
The implemented exergame system at Dream Tree Children Education Center, depicting each study phase: (A) rapport building, (B) engagement labeling and monitoring, (C) Fruit Sorting Run, and (D) Hazard Avoiding Ride. Gameplay video streams from (C) and (D) were transmitted in real time to the engagement labeling interface shown in (B).

Exergames were implemented at a local special education center that supports mental and physical health, as well as basic motor function development, for children with ASD. A 55-inch screen was placed in front of the players to display the exergames (Figures 6C and 6D). A wearable E4 wristband and 2 cameras were used to minimize external stimuli and accommodate hypersensitivity in children with ASD [41]. The E4 wristband was attached to the participant’s wrist and connected to the SG to monitor behavioral and physiological data streams. Two cameras were positioned at the front and back to capture the player’s behavior and record video. Building on this setup, a real-time visualization platform was developed and deployed to facilitate engagement labeling by parents during gameplay. The platform synchronized sensor data with live video and was displayed on two 12.9-inch iPad Pro tablets—one displaying a live video feed of the child, and the other presenting time-series plots visualizing variations in physiological and behavioral signals, including engagement status, accelerometer data, galvanic skin response, heart rate, skin temperature, and motion tracking.

### Procedure

To examine the practical applicability of the iteratively developed exergames, we conducted a human-participant study involving children with ASD. In line with case-oriented approaches used in ASD-related studies—where high interindividual variability necessitates detailed observation at the individual level—3 children with ASD (P1-P3), aged between 10 years and 13 years (mean 11, SD 1.3 years), were finally recruited and evaluated [42]. P4, who was originally scheduled to participate, withdrew consent. The refusal stemmed from P4’s sense of loss and emotional displacement after observing P3 (a peer identified as a rival) establishing a close and successful play interaction with the Jibo robot in a preceding session, effectively feeling that the friend (robot) had been taken. The demographic details of the participants are summarized in Table 2. All planned engagement labeling data and playtimes were collected without missing entries for the included participants.

**Table 2:** Pilot study demographics and clinical characteristics of children with autism spectrum disorder (ASD); 4 participants (P1-P4) were initially enrolled, with P4 withdrawing prior to study completion and excluded from subsequent analyses.

Participant (age in years)	Gender	Disability	K-Disability Grade^a^ (DSM-5^b^)	Rapport level^c^	Elementary school grade^d^	Communication capacity
P1 (10)	Female	ASD	G-2 (≈ L-2)	L-2	G-2	Inexpressible
P2 (13)	Male	ASD	G-2 (≈ L-2)	L-1	G-5	Inexpressible
P3 (11)	Male	ASD	G-2 (≈ L-2)	L-5	G-3	Expressible
P4 (11)	Male	ASD	G-2 (≈ L-2)	L-5	G-3	Withdrew

^a^The K-Disability Grades constituted the national clinical classification system for ASD (Republic of Korea). While structurally distinct from the DSM-5, shared criteria like the intelligence quotient (IQ) threshold (IQ<70) allow for cross-reference; specifically, Grade 2 aligns with Level 2 based on this threshold. Corresponding DSM-5 levels are included for cross-reference purposes.

^b^DSM-5: Diagnostic and Statistical Manual of Mental Disorders, Fifth Edition.

^c^Rapport level was evaluated by a therapist using a single item on a 5-point Likert scale (“strongly disagree” to “strongly agree”): “Overall, the interaction felt highly coordinated and established a positive, enjoyable connection.” This measure captures the 2 critical rapport dimensions emphasized in the Gratch et al [43] framework: coordination and connection.

^d^Korean elementary school education spans grades 1 to 6.

**Figure 7:**
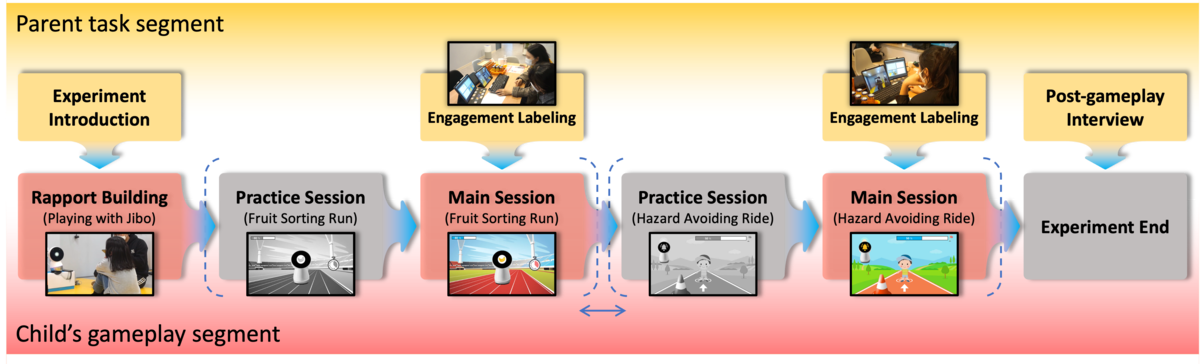
Experimental procedure across child and parent segments, depicting the full study timeline from start to end. The parent (top) and child segments (bottom) were conducted concurrently within the same session; rapport building began at experiment onset, engagement labeling was applied only during main sessions, and postgameplay interviews followed experiment completion. Back-view photos were retained as they do not permit identification; blur was applied where needed to prevent participant identification. Frames are arranged left to right to show the temporal progression; each game session (practice + main) followed a counterbalanced order across participants.

Before gameplay, participants were introduced to the exergames and informed of their respective roles (Figure 7). A preliminary session was conducted to facilitate rapport building (Figure 6A). During this session, participants interacted with Jibo, a physically embodied robot operated by the experimenter using a Wizard-of-Oz protocol. Children engaged in interactive play with the robot, which included head tilting to navigate a spaceship and responding to movement prompts (eg, dancing or playing guitar), involving audiovisual and physical interaction. The main session began after the therapist confirmed that sufficient engagement and rapport had been established. A practice session followed to assess the child’s readiness to engage with the exergames. Children who were able to follow the game flow—either independently or as determined by the therapist—proceeded to the main game session. The 2 exergames were presented in a counterbalanced order to ensure equitable exposure. The child participated in both games while each child’s parent, located in a separate room, labeled engagement status using a binary scheme (not engaged=0, engaged=1) based on real-time observation (Figure 6B) [44]. Parents were instructed to press the “Enter” key when the child appeared engaged and to refrain from pressing otherwise, following predefined operational definitions and behavioral indicators (Table 3). Before the main session, the experimenter explained the criteria and conducted a calibration using practice examples. We adopted binary labels to reduce boundary ambiguity that can increase subjectivity in finer-grained schemes (eg, ternary or multilevel labels). If the labeler judged that the annotation was incorrect over a given time-series interval, the label assignments for that interval were revised. After the gameplay session, a parent interview was conducted to gather feedback based on proxy observations of the child’s experience [45,46].

**Table 3:** Behavioral criteria for caregiver-based engagement labeling during exergame play, including binary engagement labels, their definitions, and corresponding observable behavioral indicators used to guide labeling.

Engagement label	Definition	Behavioral Indicators
Not engaged=0	The child shows no cognitive or motor participation relevant to the exergame.	(1) Looking away from the screen or robot, (2) leaving the play area, (3) stopping movement, or (4) engaging in unrelated behaviors.
Engaged=1	The child demonstrates active participation in the gameplay task.	(1) Orienting toward the screen, (2) following prompts, (3) moving body parts according to task goals, and (4) responding to game feedback

## Results

### Overview

We analyzed the temporal trends in engagement status of children with ASD, based on labels provided by their caregivers during gameplay. As each child engaged in the game for a different time duration, we first summarized the total playtime for each child (Table 4). While P1 recorded a session of 3 minutes 13 seconds in the Hazard Avoiding Ride, the average playtime across games generally aligned with expert guidance for children with ASD. To support time-aligned comparisons of engagement across participants, the time axis for each child was normalized by dividing the elapsed time by the total game duration, resulting in a scale from 0 to 1. We then modeled changes in engagement over normalized time using generalized estimating equations (GEE) with a logistic link function to account for repeated measures within participants. Specifically, we fit a GEE logistic model with an autoregressive working correlation of order 1 (AR1), treating each participant as a clustering unit and including participant indicators to control participant-level baseline differences. Engagement status (not engaged=0, engaged=1) served as the outcome variable. Given the small number of participants, we interpret inferential statistics as exploratory and emphasize effect sizes with CIs.

**Table 4:** Play durations per participant (P1-P3) for each exergame in minutes and seconds, showing participant-level variation in playtime.

Exergame type	P1	P2	P3	Mean (SD)
Fruit Sorting Run	2 min 25 s	2 min 59 s	2 min 1 s	2 min 28 s (29 s)
Hazard Avoiding Ride	3 min 13 s	2 min 25 s	1 min 55 s	2 min 31 s (39 s)

### Engagement Trends Over Time

**Figure 8:**
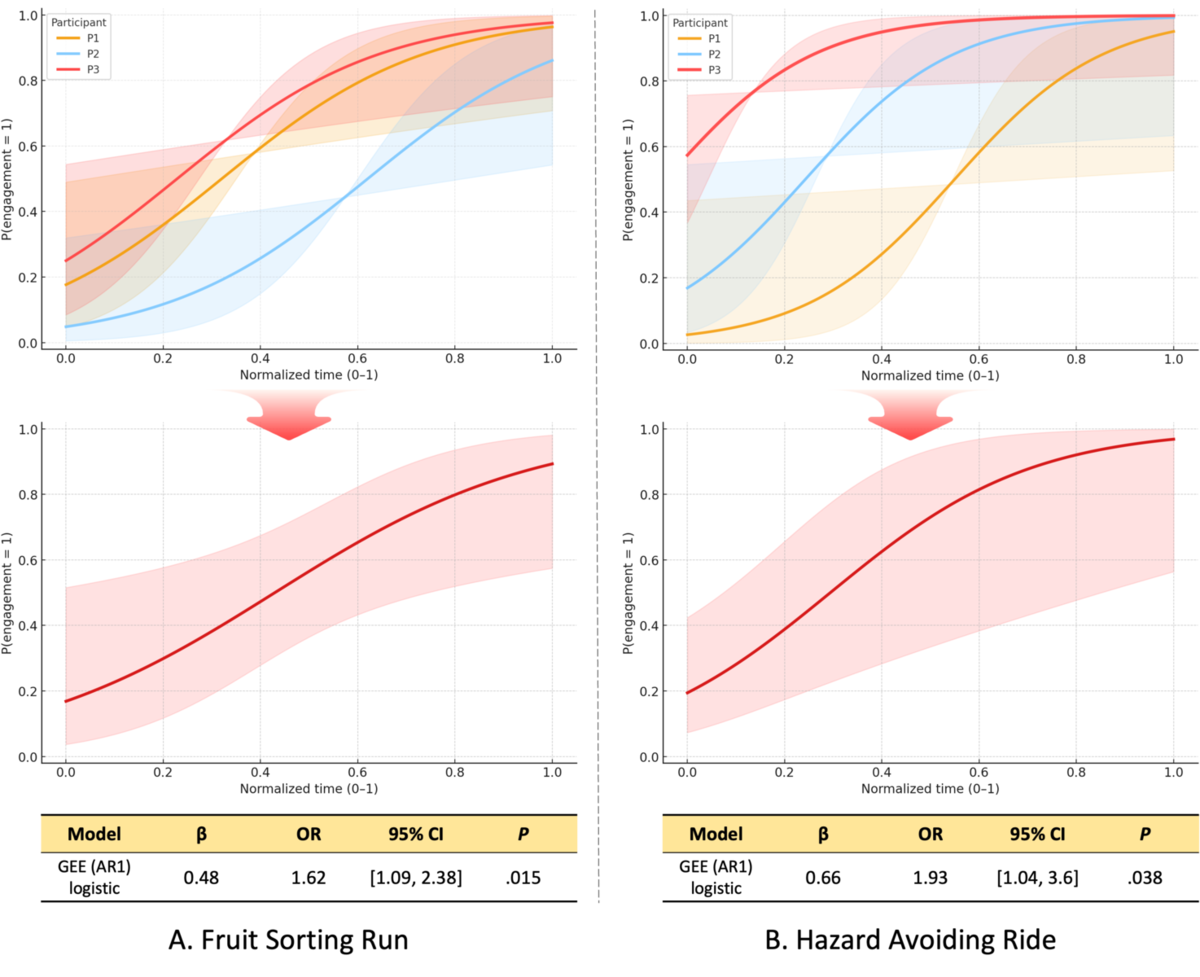
Engagement trends over normalized time (generalized estimating equations [GEE] logistic, autoregressive working correlation of order 1 [AR1]). Shaded bands indicate 95% CIs. Top: individual-level predicted probabilities shown to illustrate engagement trends. Bottom: population-averaged predicted probabilities across children with autism spectrum disorder (ASD), forming the basis for the model estimates reported in the accompanying table, with (A) Fruit Sorting Run and (B) Hazard Avoiding Ride. OR: odds ratio.

Engagement in both the Fruit Sorting Run and Hazard Avoiding Ride tended to increase over normalized time (Figure 8). Using GEE with a logistic link to account for repeated measures within participants (AR1 working correlation; participant indicators included), normalized time was significantly associated with a higher probability of engagement status =1 in the Fruit Sorting Run (per 0.1 increase: β=0.48; odds ratio 1.62, 95% CI 1.09-2.38; *P*=.02). The Hazard Avoiding Ride showed the same direction of association (per 0.1 increase: β=0.66; odds ratio 1.93, 95% CI 1.04-3.60; *P*=.04). The results present participant-specific predicted probabilities and a population-averaged trend estimated from GEE logistic models with an AR1 working correlation (95% CI). Consistent with these estimates, model-estimated P(engagement=1) tended to be higher at later normalized timepoints in both activities.

### Postinterview With Caregiver

Along with the temporal trends observed during gameplay, caregiver interviews with all 3 participating children with ASD (P1-P3) supported the observed engagement patterns. The interviews revealed distinct yet converging increases in engagement, along with perceptions of each game’s strengths and suggestions for improvement. Feedback was analyzed by game type to understand how each activity influenced engagement. In the Fruit Sorting Run, caregivers reported that participants remained attentive and engaged throughout the session. P1 appeared focused and responded positively to praise, suggesting a sense of achievement. The caregiver noted that adaptive verbal cues could further support engagement when the child was not running. P1 also showed difficulty differentiating fruits with similar colors and shapes (eg, apple vs tomato; oriental melon vs lemon), which sometimes interrupted task progression. P2 expressed a stronger preference for the Fruit Sorting Run over the Hazard Avoiding Ride. Although he showed initial hesitation, he maintained effort throughout the activity, even though he typically disengages when bored. The caregiver noted that the task aligned with his everyday preference for sorting beads by color and type, which may have supported sustained participation. Because communication was challenging, P2 relied primarily on visual prompts. P3, who usually avoids running, remained physically active without breaks and showed enjoyment. His caregiver observed that he took pride in the fruit-sorting task, which may have contributed to sustained engagement. In the Hazard Avoiding Ride, similar patterns of engagement were reported. All 3 children showed attention and engagement patterns that were consistent with an increasing trend over time, despite differences in prior experience. According to P1’s caregiver, the child focused on the task and followed the VA’s instructions, with interest increasing as the session progressed. P2, unfamiliar with bicycling, found the activity novel and remained highly concentrated; the caregiver noted that repeated exposure could promote learning. P3 was initially anxious but became increasingly engaged. Unlike in typical physical or leisure activities—where he often loses interest—he demonstrated more active and enjoyable participation once familiar with the game.

## Discussion

### Principal Results

The primary goal of this study was to design full-body serious exergames for children with ASD through an iterative, expert-informed process that emphasized both playfulness and potential functional benefits. As a result, 2 exergames were developed—Fruit Sorting Run and Hazard Avoiding Ride—each designed to promote engagement through physical activity aligned with functional skill development. To achieve this, rather than relying on a single design iteration or a pre-existing game framework, we adopted a multiphase participatory approach that integrated input from special education experts, caregivers, therapists, and HCI researchers across 4 structured phases (Figure 1). This process yielded not only 2 target exergames but also a systematic methodology that highlights the role of stakeholder involvement in tailoring game content to the cognitive, motor, and daily needs of children with ASD.

Three design considerations emerged from this process: (1) grounding gameplay in goal-oriented exercises aligned with the cognitive and motor capabilities of the target population. The exergame conceptualization was grounded in a 2×2 activity matrix informed by therapist categorization of physical and cognitive demands—a structured method that reflects prior evidence showing that tasks calibrated to users’ cognitive and motor levels support skill development and adaptive functioning in children with ASD [47]. This approach reflects SG design principles that promote aligning game tasks with users’ motor and cognitive abilities through intended in-game goals [38]. Recent studies have further shown that exercises aligned with the cognitive and motor abilities of children with ASD not only improve gross motor skills but also help motivate participation among children with ASD [48,49]. (2) Engagement scaffolding mechanisms—such as prompts and feedback from VAs—were incorporated to support sustained interaction. Existing VA-based systems for children with ASD have demonstrated the utility of contingent feedback in guiding user responses. These systems primarily focused on low-level interactions, responding to gaze [24], nonverbal conversational cues [50], or emotional expressions [51]. While such approaches support reciprocal interaction, they typically emphasize momentary cue–response dynamics rather than task-oriented guidance. Our VA expands on these foundations by delivering gameplay tasks directly, combining verbal instructions and visual cues to deliver gameplay tasks. (3) Prioritizing gameplay elements perceived by stakeholders—especially educators and caregivers—as playful, goal-oriented, and compatible with daily routines, the design approach reflects established principles in SG design for children with ASD [38]. Routine exercises that align with daily activities [52], such as jogging and cycling, can promote active engagement of children with ASD [53] and lead to improvements in skills in planning, inhibition, and cognitive flexibility, along with reductions in repetitive behaviors [54].

The pilot field study suggested that engagement trajectories tended to align with an increasing trend over time during gameplay, despite differences among participants in age, school grade, and rapport levels with the VA and occasional distraction or disengagement reflecting common attentional variability in children with ASD; behavioral labeling and caregiver interviews supported this overall pattern. Caregiver interviews indicated that even children with limited prior experience or low baseline motivation (eg, unfamiliar with bicycling [P2], typically avoidant of running, or prone to early disengagement [P3]) became more involved as they grew accustomed to the game structure. Caregivers noted that children responded positively to praise prompting (P1) and showed behavioral responses to antecedent prompting, following the VA’s instructions as interest and task focus increased during gameplay—a pattern similarly emphasized by Bernardini et al [24], who reported that children with ASD showed increased responsiveness to directive prompts delivered by a VA. Caregivers also noted that children with limited expressive communication (P1 and P2) relied more on intuitive, visual prompting; therefore, follow-up work should present options through explicit and intuitive visual media and avoid ambiguous choice boundaries. This design choice aligns with established intervention practices that commonly use visual supports, structured prompting, and reinforcement to sustain task participation [24]. Observations during gameplay further suggested that adaptive prompting, triggered in moments of hesitation or disengagement, could support sustained interaction and re-engagement [55]. Taken together, these findings highlight the VA’s role in supporting engagement through prompting during gameplay.

### Limitations and Future Directions

This study aimed to develop full-body serious exergames for children with ASD through an iterative, expert-informed design process. As an initial exploration, we tested the games with 3 children to examine engagement patterns during gameplay. Although all participants showed an upward trend in engagement, the small sample size can limit the interpretation of these patterns across the wider population of individuals with ASD. Given the heterogeneity in cognitive, motor, and behavioral characteristics, further studies with larger and more varied samples are required to examine whether similar engagement trends appear across individuals. Examining which child characteristics (eg, communication level, sensory sensitivity, motor proficiency, and baseline interest) are associated with engagement trajectories and responsiveness to prompts may be valuable for informing individualized interaction profiles. This direction may support tailoring prompt timing, modality (visual/verbal), and reinforcement style to the needs of each child rather than applying a single prompting approach. Expanding the dataset would also support future work on adaptive prompting strategies [55].

Physiological signals (eg, heart rate and skin conductance) were streamed during gameplay to assist annotators’ contextual interpretation; however, these data were not incorporated into the present analysis. As the study focused on behavioral engagement labeled by caregivers, physiological measures were treated as supplementary rather than analytic components. Future work may integrate these multimodal data to model engagement dynamics more comprehensively. In particular, combining physiological signals with gameplay context (eg, pauses, slowed pace, and errors) may help detect hesitation or overload and trigger context-sensitive prompts.

The engagement labels used a binary scheme, which reduces engagement to an on/off state and may not capture brief fluctuations or multidimensional aspects of engagement. Future work may consider multilevel or continuous labeling, along with complementary measures, to represent engagement dynamics with higher temporal resolution. Such representations can also enable evaluating how different adaptive prompting strategies affect distinct engagement components (eg, attention vs task persistence) across children.

While prompting was delivered through a VA, it was not dynamically tailored to individual behaviors. To enable context-sensitive and engagement-responsive interaction, future studies will aim to recognize engagement status in real time and apply machine learning to model and deliver adaptive prompting, aligned with O’Guinn et al [56]. Building on this direction, future work will extend the current VA-based prompting scheme from a fixed, prespecified schedule to an engagement-responsive strategy. A feasible next step is to evaluate fixed prompting versus adaptive prompting using a single-case experimental design, such as a multiple-baseline design across participants. Baseline sessions would follow the current fixed prompting logic, whereas intervention phases would introduce adaptive prompting that adjusts timing and modality based on predefined behavioral triggers observable in the existing system (eg, sustained disengagement, repeated errors, or prolonged response latency). Engagement trajectories, recovery from disengagement, and prompt responsiveness would be compared within individuals across phases, enabling rigorous individual-level evaluation under substantial interindividual variability in children with ASD.

Finally, although increased engagement was observed, the study did not assess therapeutic or educational outcomes. Future research should investigate whether these exergames can support specific intervention goals, such as improving emotional regulation, reducing stereotyped behaviors, or enhancing functional skills [57]. If such outcomes are demonstrated, the system may also offer practical value to special educators seeking to promote active participation in school-based or clinical settings. To situate the system within the broader intervention landscape, future studies will test deployment across common contexts (school-based sessions, clinical programs, and home practice) and assess feasibility factors relevant to practitioners (eg, setup time, supervision demands, and alignment with individualized goals).

### Conclusions

This study presented the iterative design and initial field evaluation of 2 full-body serious exergames for children with ASD, developed through a multiphase, expert-informed process involving 21 professionals from special education, APE, and HCI. Grounded in goal-directed physical activity and supported by engagement scaffolding mechanisms such as VA-based prompting, the exergames were tailored to promote interaction aligned with developmental needs. The design process was shaped by 3 core considerations: aligning gameplay with children’s cognitive and motor capabilities, incorporating scaffolding mechanisms to support engagement, and ensuring playfulness and perceived developmental value, such as supporting daily living skills.

Beyond prior SG approaches that primarily relied on discrete gestures or in-place interactions, this study advances exertion-intensive, whole-body exergame design by embedding sustained physical activity within structured gameplay, shaped through a series of expert collaborations. As an exploratory multiple-case pilot, we tested the 2 developed exergames with 3 children with ASD to examine feasibility and engagement patterns during gameplay, observing increasing engagement trends over time despite inter-individual differences.

By integrating expert-driven design with case-based field evaluation conducted in a special education setting, this work contributes a grounded design and evaluation framework for developing exertion-intensive exergames for children with ASD. This contribution extends prior work on serious exergames for children with ASD by demonstrating how therapist-informed exercises and structured prompting strategies can be systematically translated into playable, full-body game mechanics. While these observations remain preliminary, they provide initial indications that should be examined and strengthened with larger samples. Taken together, this exploratory multiple-case pilot provides an initial basis for a testable hypothesis that full-body serious exergames, paired with VA-based prompting, may be associated with increasing engagement over time, which should be examined in larger samples.

## Abbreviations

APE: adapted physical education
AR1: autoregressive working correlation of order 1
ASD: autism spectrum disorder
GEE: generalized estimating equations
HCI: human-computer interaction
SG: serious game
VA: virtual agent

## Data Availability

The data that support the findings of this study are available from the corresponding author upon reasonable request.

## References

[1] Mazurek MO (2013). Social media use among adults with autism spectrum disorders. Comput Hum Behav.

[2] Stanish H, Curtin C, Must A, Phillips S, Maslin M, Bandini L (2015). Enjoyment, barriers, and beliefs about physical activity in adolescents with and without autism spectrum disorder. Adapt Phys Activ Q.

[3] Boerner KE, Pearl-Dowler L, Holsti L, Wharton M, Siden H, Oberlander TF (2023). Family perspectives on in-home multimodal longitudinal data collection for children who function across the developmental spectrum. J Dev Behav Pediatr.

[4] Phillips JM, Uljarević M, Schuck RK, Schapp S, Solomon EM, Salzman E (2019). Development of the stanford social dimensions scale: initial validation in autism spectrum disorder and in neurotypicals. Mol Autism.

[5] Plötz T, Hammerla N, Rozga A, Reavis A, Call N, Abowd GD (2012). Automatic assessment of problem behavior in individuals with developmental disabilities.

[6] McCoy SM, Jakicic JM, Gibbs BB (2016). Comparison of obesity, physical activity, and sedentary behaviors between adolescents with autism spectrum disorders and without. J Autism Dev Disord.

[7] Choi H, Kim JH, Yang HS, Kim JY, Cortese S, Smith L (2024). Pharmacological and non-pharmacological interventions for irritability in autism spectrum disorder: a systematic review and meta-analysis with the GRADE assessment. Mol Autism.

[8] Mazurek MO, Engelhardt CR, Clark KE (2015). Video games from the perspective of adults with autism spectrum disorder. Comput Hum Behav.

[9] Silva GM, Souto JJDS, Fernandes TP, Bolis I, Santos NA (2021). Interventions with serious games and entertainment games in autism spectrum disorder: a systematic review. Dev Neuropsychol.

[10] Carneiro T, Carvalho A, Frota S, Filipe MG (2024). Serious games for developing social skills in children and adolescents with autism spectrum disorder: a systematic review. Healthcare (Basel).

[11] Lussier-Desrochers D, Massé L, Simonato I, Lachapelle Y, Godin-Tremblay V, Lemieux A (2023). Evaluation of the effect of a serious game on the performance of daily routines by autistic and ADHD children. Adv Neurodev Disord.

[12] Harbin SG, Davis CA, Sandall S, Fettig A (2021). The effects of physical activity on engagement in young children with autism spectrum disorder. Early Childhood Educ J.

[13] Finkelstein S, Nickel A, Barnes T, Suma E (2010). Astrojumper: designing a virtual reality exergame to motivate children with autism to exercise.

[14] Takahashi I, Oki M, Bourreau B, Suzuki K (2018). Designing interactive visual supports for children with special needs in a school setting.

[15] Graham TCN, King N, Coo H, Zabojnikova P, Gurd BJ, Samdup D (2022). Design and evaluation of an exergaming system for children with autism spectrum disorder: the children’s and families’ perspective. Front Virtual Real.

[16] Nonnis A, Bryan-Kinns N (2019). Mazi: tangible technologies as a channel for collaborative play.

[17] Soysa AI, Al MA (2020). Tangible play and children with ASD in low-resource countries: a case study.

[18] Zhiglova Y (2018). The interactive carpet—smart textile interface for children on autism spectrum disorder.

[19] Malihi M, Nguyen J, Cardy RE, Eldon S, Petta C, Kushki A (2020). Short report: evaluating the safety and usability of head-mounted virtual reality compared to monitor-displayed video for children with autism spectrum disorder. Autism.

[20] Rudovic O, Lee J, Dai M, Schuller B, Picard RW (2018). Personalized machine learning for robot perception of affect and engagement in autism therapy. Sci Robot.

[21] Rudovic O, Utsumi Y, Lee J, Hernandez J, Castelló Ferrer E (2018). CultureNet: a deep learning approach for engagement intensity estimation from face images of children with autism.

[22] Telisheva Z, Zhanatkyzy A, Turarova A, Rakhymbayeva N, Sandygulova A (2020). Automatic engagement recognition of children within robot-mediated autism therapy.

[23] Scassellati B, Boccanfuso L, Huang C, Mademtzi M, Qin M, Salomons N (2018). Improving social skills in children with ASD using a long-term, in-home social robot. Sci Robot.

[24] Bernardini S, Porayska-Pomsta K, Smith TJ (2014). ECHOES: an intelligent serious game for fostering social communication in children with autism. Inf Sci.

[25] Razavi SZ, Ali MR, Smith TH, Schubert LK, Hoque ME (2016). The LISSA virtual human and ASD teens: an overview of initial experiments.

[26] Bhattacharya A, Gelsomini M, Pérez-Fuster P (2015). Designing motion-based activities to engage students with autism in classroom settings.

[27] Bossavit B, Parsons S (2016). “This is how I want to learn”: high functioning autistic teens co-designing a serious game.

[28] Caro K, Tentori M, Martinez-Garcia AI, Alvelais M (2017). Using the FroggyBobby exergame to support eye-body coordination development of children with severe autism. Int J Hum‑Comput Stud.

[29] Peña O, Cibrian FL, Tentori M (2021). Circus in Motion: a multimodal exergame supporting vestibular therapy for children with autism. J Multimodal User Interfaces.

[30] Kosmas P, Ioannou A, Retalis S (2018). Moving bodies to moving minds: a study of the use of motion-based games in special education. TechTrends.

[31] Nicholson H, Kehle TJ, Bray MA, Heest JV (2010). The effects of antecedent physical activity on the academic engagement of children with autism spectrum disorder. Psychol Sch.

[32] Neely L, Rispoli M, Gerow S, Ninci J (2015). Effects of antecedent exercise on academic engagement and stereotypy during instruction. Behav Modif.

[33] Serino M, Cordrey K, McLaughlin L, Milanaik RL (2016). Pokémon Go and augmented virtual reality games: a cautionary commentary for parents and pediatricians. Curr Opin Pediatr.

[34] Kim B, Lee D, Min A, Paik S, Frey G, Bellini S (2020). PuzzleWalk: a theory-driven iterative design inquiry of a mobile game for promoting physical activity in adults with autism spectrum disorder. PLoS One.

[35] Graham TCN, King N, Coo H, Zabojnikova P, Gurd BJ, Samdup D (2022). Design and evaluation of an exergaming system for children with autism spectrum disorder: the children’s and families’ perspective. Front Virtual Real.

[36] (2012). Innovating for People: Handbook of Human-Centered Design Methods.

[37] Gamage V, Ennis C (2018). Examining the effects of a virtual character on learning and engagement in serious games.

[38] Whyte EM, Smyth JM, Scherf KS (2015). Designing serious game interventions for individuals with autism. J Autism Dev Disord.

[39] Ali S, Abodayeh A, Dhuliawala Z, Breazeal C, Park HW (2025). Towards inclusive co-creative child-robot interaction: can social robots support neurodivergent children’s creativity.

[40] Gilson CB, Carter EW (2016). Promoting social interactions and job independence for college students with autism or intellectual disability: a pilot study. J Autism Dev Disord.

[41] Baron-Cohen S, Ashwin E, Ashwin C, Tavassoli T, Chakrabarti B (2009). Talent in autism: hyper-systemizing, hyper-attention to detail and sensory hypersensitivity. Philos Trans R Soc Lond B Biol Sci.

[42] Kim S, Jo E, Ryu M, Cha I, Kim YH, Yoo H, Hong H (2019). Toward becoming a better self: understanding self-tracking experiences of adolescents with autism spectrum disorder using custom trackers.

[43] Gratch J, Wang N, Gerten J, Fast E, Duffy R (2007). Creating rapport with virtual agents.

[44] Jain S, Thiagarajan B, Shi Z, Clabaugh C, Matarić MJ (2020). Modeling engagement in long-term, in-home socially assistive robot interventions for children with autism spectrum disorders. Sci Robot.

[45] Boyd LE, Gupta S, Vikmani SB, Gutierrez CM, Yang J, Linstead E, Hayes GR (2018). VRSocial: toward immersive therapeutic VR systems for children with autism.

[46] Kim W, Seong M, Kim KJ, Kim S (2024). Engagnition: a multi-dimensional dataset for engagement recognition of children with autism spectrum disorder. Sci Data.

[47] Suárez-Manzano S, Ruiz-Ariza A, de Loureiro NEM, Martínez-López EJ (2024). Effects of physical activity on cognition, behavior, and motor skills in youth with autism spectrum disorder: a systematic review of intervention studies. Behav Sci (Basel).

[48] Castaño PRL, Suárez DPM, González ER, Robledo-Castro C, Hederich-Martínez C, Cadena HPG (2024). Effects of physical exercise on gross motor skills in children with autism spectrum disorder. J Autism Dev Disord.

[49] Xing Y, Wu X (2025). Effects of motor skills and physical activity interventions on motor development in children with autism spectrum disorder: a systematic review. Healthcare (Basel).

[50] Ali M, Razavi SZ, Langevin R, Mamun AI, Kane B, Rawassizadeh R, Schubert LK, Hoque E (2020). A virtual conversational agent for teens with autism spectrum disorder: experimental results and design lessons.

[51] Mower E, Black MP, Flores E, Williams M, Narayanan S (2011). Rachel: design of an emotionally targeted interactive agent for children with autism.

[52] Kern L, Koegel RL, Dyer K, Blew PA, Fenton LR (1982). The effects of physical exercise on self-stimulation and appropriate responding in autistic children. J Autism Dev Disord.

[53] Anderson-Hanley C, Tureck K, Schneiderman RL (2011). Autism and exergaming: effects on repetitive behaviors and cognition. Psychol Res Behav Manag.

[54] Fang Q, Aiken CA, Fang C, Pan Z (2019). Effects of exergaming on physical and cognitive functions in individuals with autism spectrum disorder: a systematic review. Games Health J.

[55] Kim W, Seong M, DelPreto J, Matusik W, Rus D, Kim S (2024). Exploring potential application areas of artificial intelligence-infused system for engagement recognition.

[56] O’Guinn KN, Akers J, Gerencser K (2023). Interventions targeting interactive play in individuals with autism: a systematic review. Rev J Autism Dev Disord.

[57] Meindl JN, Delgado D, Casey LB (2020). Increasing engagement in students with autism in inclusion classrooms. Child Youth Serv Rev.

